# mHealth and Application Technology Supporting Clinical Trials: Today’s Limitations and Future Perspective of smartRCTs

**DOI:** 10.3389/fonc.2017.00037

**Published:** 2017-03-13

**Authors:** Marco M. E. Vogel, Stephanie E. Combs, Kerstin A. Kessel

**Affiliations:** ^1^Department of Radiation Oncology, Technische Universität München (TUM), Munich, Germany; ^2^Institute for Innovative Radiotherapy, Helmholtz Zentrum München, Neuherberg, Germany

**Keywords:** clinical trials, app, smartRCT, eHealth, mHealth

## Abstract

Nowadays, applications (apps) for smartphones and tablets have become indispensable especially for young generations. The estimated number of mobile devices will exceed 2.16 billion in 2016. Over 2.2 million apps are available in the Google Play store^®^, and about 1.8 million apps are available in the Apple App Store^®^. Google and Apple distribute nearly 70,000 apps each in the category Health and Fitness, and about 33,000 and 46,000 each in medical apps. It seems like the willingness to use mHealth apps is high and the intention to share data for health research is existing. This leads to one conclusion: the time for app-accompanied clinical trials (smartRCTs) has come. In this perspective article, we would like to point out the stones put in the way while trying to implement apps in clinical research. Further, we try to offer a glimpse of what the future of smartRCT research may hold.

## Introduction

In the twenty-first century, digitalization in day-to-day life is ubiquitous, and besides conventional computers, laptops, and mobile phones, the use of smartphones is continuously increasing and far beyond writing messages or phone calls. Applications (apps) for smartphones and tablets have become indispensable, especially for young generations; however, increasing use of apps in the middle-aged and elderly population is observed, thus arguing for a common use across generation borders (1). The estimated number of mobile devices will exceed 2.16 billion in 2016 (2). Over 2.2 million apps are available in the Google Play store^®^, and about 1.8 million apps are available in the Apple App Store^®^. Google and Apple distribute nearly 70,000 apps each in the category Health and Fitness, and about 33,000 and 46,000 each in medical apps (3, 4). The WHO defines these tools under the label “mHealth” or “eHealth” as “medical and public health practice supported by mobile devices, such as mobile phones, patient monitoring devices, personal digital assistants, and other wireless devices” (5). The willingness to use mHealth apps or devices seems high. In a current study, Chen et al. (6) showed a great acceptance (77%) to share data for health research, which leads to the natural conclusion: the time for app-accompanied clinical trials has come. In addition, we are living in the era of Big Data, where lots of data are generated and analyzing strategies need to be found. Big Data is defined as “high volume, high velocity, and/or high variety information assets that require new forms of processing to enable enhanced decision making, insight discovery, and process optimization.” (7)

Currently, the medical field shows an evolving trend in developing apps used as tools for behavior change therapy or lifestyle intervention, such as diabetes, weight loss, or exercise performance (8–10). But to date, only a few researchers like Volkova et al. (11) use mobile apps as supporting tools in trials. In their research, they launched the app-based Food Label Trial investigating the impact of labels on consumer behavior. They created the very apt term “smartRCTs” (app-accompanied randomized controlled trials).

In this perspective article, we would like to investigate the rocky path while attempting to implement apps in clinical research. Further, we try to offer a glimpse of what the future of smartRCT research may hold.

## Limitations and Barriers of smartRCTs

### Legal Limitations—Do Researchers Need a Law Degree?

When implementing apps in clinical research, one of the highest barriers to overcome is the legal limitations. The requirements differ in each country. We describe the situation in Germany, as it has one of the strictest data privacy regulations [place 7 in Privacy and Human Rights Report 2007 (12)], and we are experienced with the legal situation. Clinical trials need to be approved by the ethics committee (preventing unethical practice) and the data protection officer (enforcing data privacy laws). Usually, the ethics commission supports smartRCTs, if all the data privacy regulations are met (13). However, it is difficult to meet the requirements of the data privacy officer. Informal consent by patients must be obtained before entering the trial. This means, besides the usual trial information, patients have to be educated about the app technology, secure data transfer (anonymous or pseudonymous), data storage, time of storage, and the possibility to delete data if they discontinue the study. When recruiting patients prospectively during treatment this is less problematic, but gaining patient consent by phone or letter is difficult and time consuming.

The type of data transfer needs to be chosen carefully: anonymous data transfer (no patient-related data are transferred) will not conflict with data privacy regulations. Whereas pseudonymous data transfer (patient data are tagged with a pseudonym) requires proper planning. Pseudonyms must be generated to be untraceable and securely stored. The best approach is to set up two different servers: on server A, patients’ data are stored, while server B contains the pseudonym and identifier. Both servers are not connected, hence, only authorized trial personal, who has access to both servers, is able to retrieve sensitive data (14).

Further, it is important to be in full control of patient data, as the right to informational self-determination is fundamental. Data deletion based on patient’s wish needs to be possible. However, services from third-party providers often store data on remote servers outside the respective law system. Complete deletion of data is impossible and, therefore, such services do not comply with the law. Furthermore, secure data storage must be ensured for at least 10 years, which might lead to data overload (described below). It is important to stay—already in the phase of trial planning—in close contact with the ethics commission and the data privacy officer to meet all regulations and adapt the study protocol, if necessary. It would be beneficial if the German data privacy regulations and laws would get adjusted to the new technology to reduce bureaucracy and allow for high-tech research.

Medical apps demand a different quality standard. It is crucial that apps in smartRCTs need to meet standardized criteria, as they are involved in patient care. In Germany, apps that are considered as medical products need to comply with the Medizinproduktegesetz (Law for Medical Products) and thus must meet stricter criteria as common apps. Medical products need clinical testing and certification. Further, compliance with the Telemedizingesetz (Law for Telemedicine) needs to be ensured. The U.S. Food and Drug Administration published similar guidelines (15). Hence, it is viable to ensure the legal status of the app before starting the programming phase.

### Patients—An App a Day Keeps the Doctor Away?

Limitations of smartRCTs are set by the patients themselves. In earlier publications, we investigated the attitude toward apps for therapeutic and scientific purposes. We saw a dependency on age and gender. Men and participants <60 years are more likely to use an app (16). The reluctance of female patients needs to be further evaluated. The age appears to be a limiting factor in app use. In 2014, only 18% of elderly people (>65 years) owned smartphones or tablets. Seventy-seven percent of the elderlies would request help when learning to use this new technology (17). Consequentially, this may lead to a lack of compliance when using apps in smartRCTs. In contrast, Smith et al. (18) showed a growing trend in smartphone use by people >65 years in 2015: already 27% in the U.S. owned smartphones, which means older citizens increasingly adapt to the new technology. Either way, smartRCTs must be planned considering involved patient cohort. Trials with elderly participants need careful selection of the mobile tools and possibly intense pretrial participant teaching. After all, Denis et al. showed that patient using mHeatlh apps feel closer to their treating doctor due to better communication (19).

Another problem could be that patients do not always own an adequate mobile device. Although in industrial countries like Germany (60%) or the United States (72%) a majority of the citizens is in possession of a mobile device (20), the variety of devices and operating systems is huge. Certainly, participation in smartRCTs can be predicated by adding smartphone possession (maybe even with certain operating systems) to the inclusion criteria, but this leads to preselection and therefore to biased results. One approach is to hand out a mobile device with a specified operating system to the participants while taking part in the trial. This reduces the costs for developing apps for different operating systems and ensures non-biased samples, however, requires funding to purchase devices. In summary, patients might be a limiting factor when launching a smartRCT, but proper preparation and careful planning can lead to an exceptional trial compliance.

### Staff—An Important Cog in the Wheel?

As in the case with patients, the age of clinicians and principal investigators is an obstacle when implementing smartRCTs. In an earlier series, we investigated the attitude of health-care professionals toward app use of patients during treatment and aftercare. The idea of implementing apps was supported by 84.3%; 64.8% even preferred to be alerted if patients enter severe side effects, which require action. The majority (93.5%) supports scientific evaluation of the collected data. The named arguments against app use were legal uncertainty regarding medical responsibility; wish for sole personal contact between health-care professionals and patient; missing technical skills; and lack of time (14).

Legal uncertainty should be minimal within smartRCTs, as the study protocol describes precise treatment algorithms, which are audited by the ethics commission. Principal investigators and staff need to be technically trained. Without proper skills, they are not able to work with the used tools, as well as teach patients. Certainly, the fear of additional work exists. However, Denis et al. (19) showed that treating doctors needed less than 15 min per week for data analysis and phone calls within the entire cohort of 42 patients. With the appropriate development of automated data analyses, the staffs’ work time can be minimized. Time-consuming clinical visits might be reduced, as some are replaced by app-based follow-ups. The delegation of tasks to specifically trained medical support staff like study nurses could reduce high workloads for principal investigators.

As always when introducing new technologies, there are supporters and critics within the medical profession. However, a smartRCT can only be successful if all principal investigators and the participating staff are technically educated and ready to support the project. Otherwise, failure is unavoidable.

### Technical Realization—Let Us Start Programming in the Garage?

When overcoming all barriers, technical realization of smartRCTs is a minor obstacle. Programming from scratch needs qualified staff, which increases the costs of trials. Projects with apps require also medical computer scientists who not only provide technical skills but also understand how medical trials are conducted and have basal knowledge of the physicians’ daily work and patients’ needs. However, there are open source development kits offered by providers (e.g., Apple Researchkit^®^ and Google Study kit^®^) to compose an app based on the respective operating system. For instance, Bot et al. (21) use the Apple Researchkit^®^ in his “mPower” Parkinson trial.

As stated above, it is important to match the legal requirements for data transfer, security, and privacy when developing apps. Data transfer to a cloud or server needs to be encrypted to ensure the best possible protection of highly sensitive patient information. He et al. (22) showed that few Android mHealth apps match these criteria, although technical capabilities are already established: Thilakanathan et al. (23) developed a secure protocol for sharing patient data in clouds, and Silva et al. (24) presented the DE4MHA algorithm for secure encryption. Moreover, data storage needs to be encrypted to protect patient data from unauthorized access. A long-term storage on secure servers in favor of transparency and plausibility of trial data is needed. This may lead to higher costs and data overload. Today, public health studies need already data storage capacity of 10 trillion bytes (10 TB) and more. This would equal tens of millions of floppy disks (25). Eventually, this is a future challenge for data transfer, storage, and management systems, which is no insurmountable problem.

## Future Perspective of smartRCTs

### Apps Supporting Clinical Trials—Time Is Money

Besides all the barriers, smartRCTs hold numerous benefits in supporting trials. Khan et al. (26) observed the work time of three individual clinical trial managers and showed that tasks such as documentation (24%), administrative work (20%), and recruitment (16%) are time consuming. Moreover, activities, such as filling out case report forms (12%), data entry (10%), and recruiting eligible patients (9%), are exhausting. The most commonly used tool was paper (24%). Data collection with apps can reduce all those tasks and thus duration. Neuer et al. (27) suggest a 30% decrease in duration by using electronic data capture. Therefore, data analyses can be achieved faster, and results are quicker implemented into clinical routine (28).

**Figure 1:**
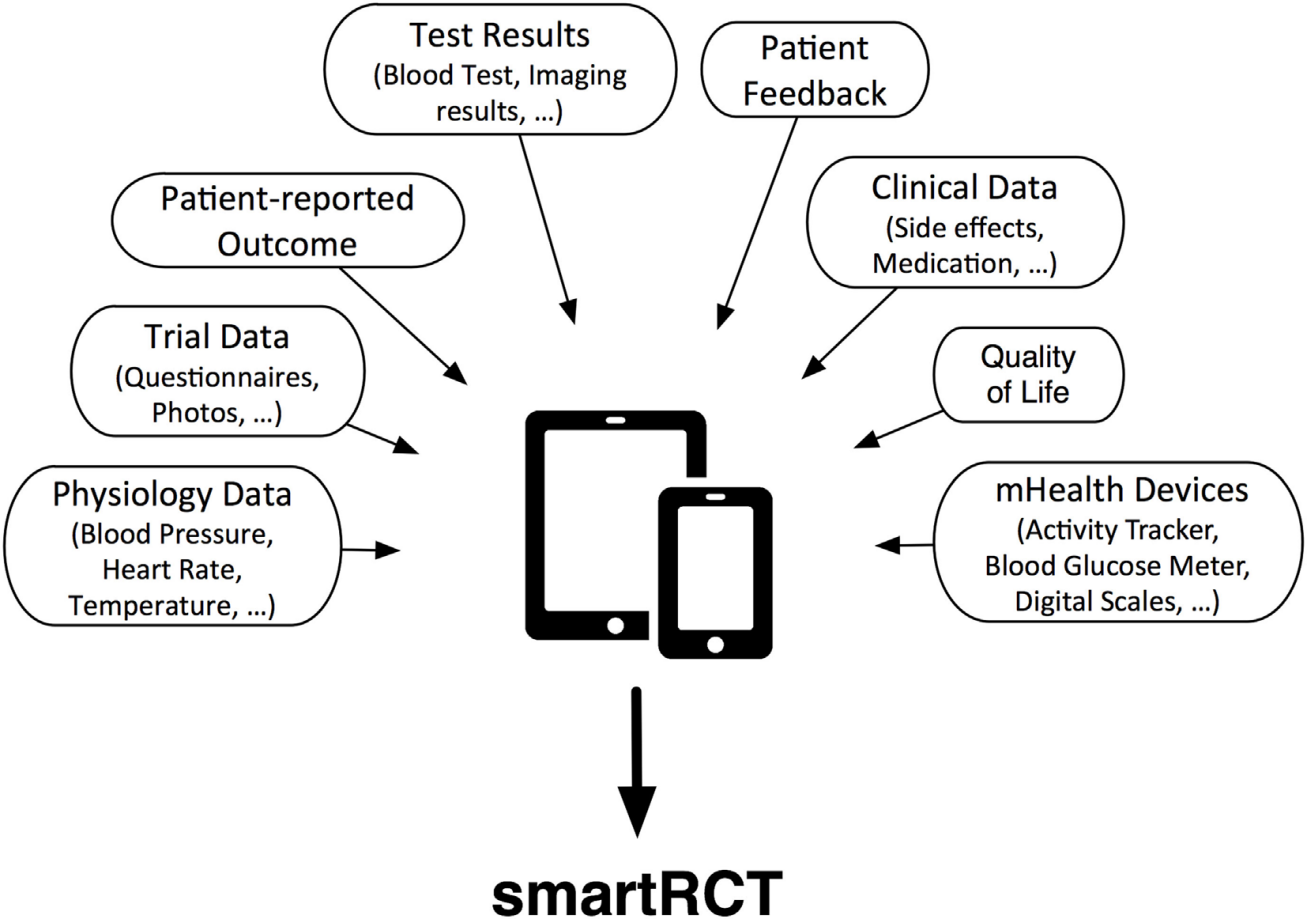
**Data collection by mobile applications for smartRCTs**.

The whole process of documentation could be simplified by asking patients to document trial parameters, quality of life scores, or other information *via* an app. Consequentially, a new dimension of information is added to the usual trial data: the patients’ view. Going even a step further, mHealth devices such as activity trackers, blood pressure monitors, blood glucose meters, or personal scales can be connected with apps. Hence, the completeness and timeliness of the data are increased, because the course of the disease is monitored longitudinally and not only cross-sectional as it is the case with classical periodical visits. Highly compliant patients could even enter blood test or imaging results made by other physicians (see Figure 1).

Needless to say, it is possible to develop apps to be used two sided, and physicians or study nurses could also enter data into the app. Standardized entry is guaranteed, and paperwork is diminished. Reduced paperwork protects the environment and leads to a more secure archiving of patient data. Subsequent changes of data are more difficult; hence, data transparency is improved. Errors during processing and digitizing data are prevented. Dependencies can be used to check entries for plausibility, and automated algorithms can verify inputs already before storing.

The time-consuming recruitment process can be simplified by using apps. It is difficult to find eligible patients for trials using the traditional form of recruitment. With mHealth tools, a wide range of people can be approached, and, therefore, it is easier, faster, and more cost-effective. Laws et al. (29) showed that online recruitment compared to a practitioner and face-to-face recruitment is the quickest and cheapest form with average costs of AUD$ 14 per participant.

With all named benefits a higher cost-effectiveness can be achieved. Sertkaya et al. (30) applied a media data study and showed total costs of $78.6 million for phase 1–4 trials investigating drugs in oncology. Administrative staff costs (phase 3: 20.40%), physician costs (phase 3: 7.08%), source data verification costs (phase 3: 3.52%), patient recruitment costs (phase 3: 2.71%), and data management costs (phase 3: 0.34%) (30) can be reduced by using app technology where appropriate. The prerequisite is that apps operate as safe and without patient discomfort as humans. Sertkaya et al. suggest a total cost reduction of 7.91% (=$6.2 million) if mobile technology is used. Eisenstein et al. (31) calculated a reduction of 9.8% by using electronic data capture.

A huge problem within trials is the inevitable subjectivity of research personnel. Various studies of Hróbjartsson et al. (32–34) showed an occurring bias, especially if blinding is not feasible. App-based data collection is objective as well as standardized, for example, pre-validated questionnaires can be used. Furthermore, smartRCTs simplify multicenter trials as data transfer is easier and all investigators are closely connected. It is possible to centralize the data storage and prevent data loss (28). Moreover, app-based study procedures are equal and objective within all centers.

The mentioned advantages can be of great scientific value in radiation oncology as it is a highly technical discipline. In contrast to classical clinical visits, apps can be used for trial documentation of side effects caused by radiotherapy or concomitant radio-chemotherapy. Trial compliance is enhanced as apps can be used to remind patients of radiation dates or drug intake. Continuous aftercare over years plays an important role in radiation oncology. App-based research could enhance documentation and therefore simplify trials on long-term toxicity. The implementation of smartRTCTs would be of great benefit as it marks the departure into the new era of radiation oncology 4.0 (35).

### Apps and Big Data—I Have a Dream …

smartRCTs and app-based research generate a huge amount of data—Big Data. Only a fraction of collected trial data are used and published. The data could, however, be utilized to perform sub-studies or can be merged to acquire new insights for clinical day-to-day life. Epidemiological researchers could identify patterns, causes, and effects of diseases without high costs and workload. In return, it is important to improve the level of technical skills within epidemiological studies (36).

Moreover, Big Data can be used to perform clinical trials *in silico*, which means computer simulations are run instead of classical studies (37). Less animal testing and patient recruitment in pharmaceutical or other trials would be needed (38). Especially, research in the area of highly rare conditions would benefit. Instead of using the hard road of recruiting a huge number of patients to gather valid results, computer simulation can accompany trials in rare tumor or diseases. Furthermore, Big Data shows potential in the evolving genomics research. A variety of recently published studies used electronic health data to show relationships between genetic variations and clinical conditions (39–41). Bowton et al. (42) showed this approach to be cost-effective and quick. The Omics movement led to further research disciplines: pharmacogenomics investigates effects of genetics on the individuals’ drug response. Omics can be used to predict disease probability, a great tool for preventive medicine (43). Hence, Big Data and Omics will be a major step toward personalized and precise medicine.

## Conclusion

smartRCTs and app-based studies are the future of medical research—radiation oncology in particular. While there are certain barriers—especially the data privacy laws—the advantages outweigh the limitations. It would be desirable if politicians and lawmakers establish better opportunities and adjust the regulations to the new technology. This is possible without undermining the right to informational self-determination and data privacy. Further, all parties involved—data privacy officers, ethical commission, patients, and researchers—need to support an aspired smartRCT. Necessarily, apps for research need to meet certain criteria concerning patient safety and good clinical practice; therefore, generally accepted standards for trial apps need to be established. If so, apps can reduce trial costs, study duration, and subjectivity bias as well as collect a wider range of data. One thing is clear: smartRCT is not a question of whether or not, but of when and how.

## Author Contributions

MV wrote the manuscript. SC advised and edited this manuscript. KK advised and edited the manuscript and proposed the initial concept. All authors approved the manuscript.

## Conflict of Interest Statement

The authors declare that the research was conducted in the absence of any commercial or financial relationships that could be construed as a potential conflict of interest.

## References

[1] Ehlers DK Huberty J Buman M Hooker S Todd M de Vreede GJ. A novel inexpensive use of smartphone technology for ecological momentary assessment in middle-aged women. J Phys Act Health (2016) 13(3):262–8. 10.1123/jpah.2015-0059 26284689

[2] eMarketer. 2 Billion Consumers Worldwide to Get Smart (Phones) by 2016. (2014). Available from: http://www.emarketer.com/Article/2-Billion-Consumers-Worldwide-Smartphones-by-2016/1011694 (Archived by WebCite^®^ at http://www.webcitation.org/6jGNrtCR8)

[3] Jordan J App Store Metrics. (2016). Available from: http://www.pocketgamer.biz/metrics/app-store/ (Archived by WebCite^®^ at http://www.webcitation.org/6jGO48VjD)

[4] Maurer U Vogelzang M Android Statistics. (2016). Available from: http://www.appbrain.com/stats (Archived by WebCite^®^ at http://www.webcitation.org/6jGO1n8c5)

[5] World Health Organization (WHO). mHealth: New Horizons for Health Through Mobile Technologies: Second Global Survey on eHealth. Geneva (2011).

[6] Chen J Bauman A Allman-Farinelli M. A study to determine the most popular lifestyle smartphone applications and willingness of the public to share their personal data for health research. Telemed J E Health (2016) 22:655–65. 10.1089/tmj.2015.0159 26958742

[7] Beyer MA Laney D The Importance of ‘Big Data’: A Definition. Stamford, CT: Gartner (2012). p. 2014–8.

[8] Gimenez-Perez G Recasens A Simo O Aguas T Suarez A Vila M Use of communication technologies by people with type 1 diabetes in the social networking era. A chance for improvement. Prim Care Diabetes (2016) 10(2):121–8. 10.1016/j.pcd.2015.09.002 26428527

[9] Stephens JD Yager AM Allen J. Smartphone technology and text messaging for weight loss in young adults: a randomized controlled trial. J Cardiovasc Nurs (2017) 32(1):39–46. 10.1097/JCN.0000000000000307 26646593 PMC4896848

[10] Fong SS Ng SS Cheng YT Zhang J Chung LM Chow GC Comparison between smartphone pedometer applications and traditional pedometers for improving physical activity and body mass index in community-dwelling older adults. J Phys Ther Sci (2016) 28(5):1651–6. 10.1589/jpts.28.1651 27313391 PMC4905930

[11] Volkova E Li N Dunford E Eyles H Crino M Michie J “Smart” RCTs: development of a smartphone app for fully automated nutrition-labeling intervention trials. JMIR Mhealth Uhealth (2016) 4(1):e23. 10.2196/mhealth.5219 26988128 PMC4816928

[12] Privacy and Human Rights 2006: An International Survey of Privacy Laws and Developments. Washington, DC: Electronic Privacy Information Center & Privacy International (2007).

[13] McCann M The smartphones study: an analysis of disciplinary differences in research ethics committee responses to phone app-based automated data collection. Eur J Public Health (2016) 26(Suppl):1 10.1093/eurpub/ckw164.00226370436

[14] Kessel KA Vogel MM Schmidt-Graf F Combs SE. Mobile apps in oncology: a survey on health care professionals’ attitude toward telemedicine, mHealth, and oncological apps. J Med Internet Res (2016) 18(11):e312. 10.2196/jmir.6399 27884810 PMC5146327

[15] Mobile Medical Applications – Guidance for Industry and Food and Drug Administration Staff: Food and Drug Administration. (2015). Available from: http://www.fda.gov/downloads/MedicalDevices/DeviceRegulationandGuidance/GuidanceDocuments/UCM263366.pdf (Archived by WebCite^®^ at http://www.webcitation.org/6mtiwpLTZ)

[16] Kessel KA Vogel MM Combs SE Mobile applications as part of the oncological therapy – a survey on evaluating the acceptance in a German Oncology Center. Strahlenther Onkol (2016) 192(1):143 10.1007/s00066-016-0974-z

[17] Smith A Older Adults and Technology Use: Pew Research Center. (2014). Available from: http://www.pewinternet.org/2014/04/03/older-adults-and-technology-use/ (Archived by WebCite^®^ at http://www.webcitation.org/6mqbkRuIu)

[18] Smith A U.S. Smartphone Use in 2015: Pew Research Center. (2015). Available from: http://www.pewinternet.org/2015/04/01/us-smartphone-use-in-2015/ (Archived by WebCite^®^ at http://www.webcitation.org/6nF7QSBT7)

[19] Denis F Viger L Charron A Voog E Dupuis O Pointreau Y Detection of lung cancer relapse using self-reported symptoms transmitted via an internet web-application: pilot study of the sentinel follow-up. Support Care Cancer (2014) 22(6):1467–73. 10.1007/s00520-013-1954-9 24414998

[20] Poushter J Smartphone Ownership and Internet Usage Continues to Climb in Emerging Economies: Pew Research Center. (2016). Available from: http://www.pewglobal.org/2016/02/22/smartphone-ownership-and-internet-usage-continues-to-climb-in-emerging-economies/ (Archived by WebCite^®^ at http://www.webcitation.org/6mquZnvXU)

[21] Bot BM Suver C Neto EC Kellen M Klein A Bare C The mPower study, Parkinson disease mobile data collected using ResearchKit. Sci Data (2016) 3:160011. 10.1038/sdata.2016.11 26938265 PMC4776701

[22] He D Naveed M Gunter CA Nahrstedt K. Security concerns in android mHealth apps. AMIA Annu Symp Proc (2014) 2014:645–54. 25954370 PMC4419898

[23] Thilakanathan D Calvo RA Chen S Nepal S Glozier N. Facilitating secure sharing of personal health data in the cloud. JMIR Med Inform (2016) 4:e15. 10.2196/medinform.4756 27234691 PMC4902857

[24] Silva BM Rodrigues JJPC Canelo F Lopes IC Zhou L. A data encryption solution for mobile health apps in cooperation environments. J Med Internet Res (2013) 15:e66. 10.2196/jmir.2498 23624056 PMC3636327

[25] Schnabel J Big Data – Overload: The Quest for Knowledge in an Era Flooded with Information. Baltimore: Johns Hopkins Public Health (2012). p. 20–4.

[26] Khan SA Payne PRO Johnson SB Bigger JT Kukafka R. Modeling clinical trials workflow in community practice settings. AMIA Annu Symp Proc (2006) 2006:419–23. 17238375 PMC1839500

[27] Neuer A Slezinger E Warnock N The Upfront Cost Hurdle of EDC: Applied Clinical Trials. (2010). Available from: http://www.appliedclinicaltrialsonline.com/upfront-cost-hurdle-edc (Archived by WebCite^®^ at http://www.webcitation.org/6mvSo6yeO)

[28] Kessel KA Bohn C Engelmann U Oetzel D Bougatf N Bendl R Five-year experience with setup and implementation of an integrated database system for clinical documentation and research. Comput Methods Programs Biomed (2014) 114(2):206–17. 10.1016/j.cmpb.2014.02.002 24629596

[29] Laws AR Litterbach EK Denney-Wilson AE Russell GC Taki S Ong KL A comparison of recruitment methods for an mHealth intervention targeting mothers: lessons from the growing healthy program. J Med Internet Res (2016) 18(9):e248. 10.2196/jmir.5691 27634633 PMC5043120

[30] Sertkaya A Birkenbach A Berlind A Eyraud J Examination of Clinical Trial Costs and Barriers for Drug Development Eastern Research Group/U.S. Department of Health and Human Services. (2014). Available from: https://aspe.hhs.gov/report/examination-clinical-trial-costs-and-barriers-drug-development (Archived by WebCite^®^ at http://www.webcitation.org/6mvPwSwvm)

[31] Eisenstein EL Collins R Cracknell BS Podesta O Reid ED Sandercock P Sensible approaches for reducing clinical trial costs. Clin Trials (2008) 5(1):75–84. 10.1177/1740774507087551 18283084

[32] Hrobjartsson A Thomsen AS Emanuelsson F Tendal B Hilden J Boutron I Observer bias in randomised clinical trials with binary outcomes: systematic review of trials with both blinded and non-blinded outcome assessors. BMJ (2012) 344:e1119. 10.1136/bmj.e1119 22371859

[33] Hróbjartsson A Thomsen AS Emanuelsson F Tendal B Hilden J Boutron I Observer bias in randomized clinical trials with measurement scale outcomes: a systematic review of trials with both blinded and nonblinded assessors. CMAJ (2013) 185(4):E201–11. 10.1503/cmaj.120744 23359047 PMC3589328

[34] Hrobjartsson A Thomsen AS Emanuelsson F Tendal B Rasmussen JV Hilden J Observer bias in randomized clinical trials with time-to-event outcomes: systematic review of trials with both blinded and non-blinded outcome assessors. Int J Epidemiol (2014) 43(3):937–48. 10.1093/ije/dyt270 24448109

[35] Zimmermann D On the Cusp of Medicine 4.0. (2015). Available from: http://www.healthcare-in-europe.com/en/article/15539-on-the-cusp-of-medicine-4-0.html (Archived by WebCite^®^ at http://www.webcitation.org/6oC1sKhZX)

[36] Mooney SJ Westreich DJ El-Sayed AM. Epidemiology in the era of big data. Epidemiology (2015) 26(3):390–4. 10.1097/EDE.0000000000000274 25756221 PMC4385465

[37] The power of big data must be harnessed for medical progress. Nature (2016) 539(7630):467–8. 10.1038/539467b 27882983

[38] Viceconti M Henney A Morley-Fletcher E In silico clinical trials: how computer simulation will transform the biomedical industry. Int J Clin Trials (2016) 3(2):37–46. 10.18203/2349-3259.ijct20161408

[39] Denny JC Ritchie MD Crawford DC Schildcrout JS Ramirez AH Pulley JM Identification of genomic predictors of atrioventricular conduction: using electronic medical records as a tool for genome science. Circulation (2010) 122(20):2016–21. 10.1161/CIRCULATIONAHA.110.948828 21041692 PMC2991609

[40] Kullo IJ Ding K Jouni H Smith CY Chute CG. A genome-wide association study of red blood cell traits using the electronic medical record. PLoS One (2010) 5(9):e13011. 10.1371/journal.pone.0013011 20927387 PMC2946914

[41] Ritchie MD Denny JC Crawford DC Ramirez AH Weiner JB Pulley JM Robust replication of genotype-phenotype associations across multiple diseases in an electronic medical record. Am J Hum Genet (2010) 86(4):560–72. 10.1016/j.ajhg.2010.03.003 20362271 PMC2850440

[42] Bowton E Field JR Wang S Schildcrout JS Van Driest SL Delaney JT Biobanks and electronic medical records: enabling cost-effective research. Sci Transl Med (2014) 6(234):234cm3. 10.1126/scitranslmed.3008604 24786321 PMC4226414

[43] Chen R Snyder M. Promise of personalized omics to precision medicine. Wiley Interdiscip Rev Syst Biol Med (2013) 5(1):73–82. 10.1002/wsbm.1198 23184638 PMC4154620

